# Mind-Body Medicine for Multiple Sclerosis: A Systematic Review

**DOI:** 10.1155/2012/567324

**Published:** 2012-11-22

**Authors:** Angela Senders, Helané Wahbeh, Rebecca Spain, Lynne Shinto

**Affiliations:** ^1^Department of Neurology, Oregon Health & Science University, Portland, OR 97239, USA; ^2^Department of Neurology, Portland Veterans Affairs Medical Center, Portland, OR 97239, USA

## Abstract

*Background*. Mind-body therapies are used to manage physical and psychological symptoms in many chronic health conditions. *Objective*. To assess the published evidence for using mind-body techniques for symptom management of multiple sclerosis. *Methods*. MEDLINE, PsycINFO, and Cochrane Clinical Trials Register were searched from inception to March 24, 2012. Eleven mind-body studies were reviewed (meditation, yoga, biofeedback, hypnosis, relaxation, and imagery). *Results*. Four high quality trials (yoga, mindfulness, relaxation, and biofeedback) were found helpful for a variety of MS symptoms. *Conclusions*. The evidence for mind-body medicine in MS is limited, yet mind-body therapies are relatively safe and may provide a nonpharmacological benefit for MS symptoms.

## 1. Introduction

Multiple sclerosis (MS) is a chronic neurological disorder with emotional, cognitive, and physical consequences. Patients can experience a diverse array of symptoms including impaired mobility, sensory disturbance, chronic pain, fatigue, bladder and bowel dysfunction, depression, and cognitive impairment. Patients report high levels of stress, independent of physical disability [1–3], and the risk of developing stress-related disorders like anxiety and depression is high (25% and 34–50% lifetime prevalence, resp.) [4–7]. There is no cure for MS, and both disease-modifying and symptomatic therapies have limitations in compliance due to side effects and cost [8, 9]. 

Mind-body therapies are used by a growing number of American adults and children [10]. In a survey of 1,110 MS patients, 32% of respondents had used mind-body modalities and reported a high perceived benefit [11]. The National Center for Complementary and Alternative Medicine defines mind-body therapies as those that integrate the brain, mind, body, and behavior, with the intent to use the mind to affect physical functioning and promote health [12]. Examples of such modalities include meditation, yoga, relaxation techniques, breathwork,visual imagery, hypnotherapy, and biofeedback. While these practices vary in technique and application, they share a common objective to enhance the capacity of the mind to improve physical and psychological wellbeing. 

Many mind-body therapies can be taught in a clinical setting and then subsequently used at home by the patient in a self-directed way, making them ideal tools to promote increased self-efficacy. Mind-body techniques often have a calming effect on the autonomic nervous system [13] and thus may be helpful for conditions where psychological stress is a factor. From this perspective, mind-body interventions hold strong potential for use in MS. Broad reviews have addressed the use of mind-body therapies in general practice [14] and neurology [15], yet no comprehensive review of evidence for MS exists. The objective of this paper is to systematically review the published evidence for mind-body therapies for symptom management in MS.

## 2. Methods

One reviewer performed the searches, assessed inclusion criteria, and extracted data on trial design and outcomes. Two reviewers independently assessed methodology and risk of bias for each study included in this paper. Because we expected the sample size of mind-body studies to be small, we intentionally kept inclusion criteria broad.

### 2.1. Types of Studies

We included only controlled trials in this paper. 

### 2.2. Types of Participants

We included studies of any kind of MS. Studies that did not verify diagnosis of MS or use accepted diagnostic criteria for their time were included in this paper; however, their risk of bias is included in our analysis. We added the category “MS diagnosis confirmation and criteria” to the Cochrane Risk of Bias Table for this purpose.

### 2.3. Types of Interventions

Mind-body therapies included meditation and mindfulness, yoga, hypnosis, biofeedback, relaxation, imagery, tai chi, and qi gong. 

### 2.4. Types of Outcome Measures

Included were studies with any MS outcome related to physical or psychosocial wellbeing. Specific outcomes were not defined.

### 2.5. Search Strategy for Identification of Studies

One reviewer (A. Senders) searched the electronic databases MEDLINE, PsychInfo, and Cochrane Clinical Trials Register. Databases were searched in Ovid (http://www.ovid.com/) from inception to March 24, 2012, using the exploded medical subject headings (MeSH): Mind-Body Therapies; Mind-Body Relations, Metaphysical; Therapeutics; Hypnosis; Yoga; Imagery; Biofeedback; Tai Ji; Meditation; Relaxation; Muscle Relaxation; Relaxation Therapy; Breathwork; Breathing Exercises. Tai chi, Qi Gong, Breathing Technique, and Mindfulness were searched as keywords. These MeSH terms and keywords were then combined with MS terms Multiple Sclerosis; Multiple Sclerosis, Chronic Progressive; Multiple Sclerosis, Relapsing-Remitting. A medical librarian was consulted to review the search protocol. Only studies reported in the English language were considered in this review. A manual review of references or related reviews was not completed. The specific search strategy can be found in the Appendix.

### 2.6. Selection of Studies

One author (A. Senders) conducted the initial search. Potential publications were selected by screening titles and abstracts for clinical trials that used a mind-body intervention for any type of multiple sclerosis. Final selections were made by screening full articles for the following inclusion criteria: any type of MS, any mind-body intervention, any kind of comparator group, and any outcome related to physical or mental wellbeing. 

### 2.7. Data Extraction and Management

Data extraction from each study was performed by one author (A. Senders) using a data collection form that included study design, participant characteristics, type and duration of intervention, comparator treatment, primary and secondary outcome measures, whether or not homework was prescribed, adherence to homework, dropout rate/loss to follow up, and summary of results. Authors of reviewed studies were not contacted for missing information or clarification.

### 2.8. Assessment of Risk of Bias in Included Studies

The methodological quality of clinical trials was assessed according to the Cochrane Risk of Bias Tool [36]. Assessments of bias were independently performed for each paper in duplicate (AS, HW, or LS) and any disagreements were recorded and resolved through discussion. 

### 2.9. Issues of Heterogeneity

One objective of this paper was to grade the body of evidence for mind-body medicine in MS using the Grading of Recommendations Assessment, Development and Evaluation tool (GRADE) [37]. However, a meta-analysis or reliable grading of the body of evidence is not feasible due to a large amount of clinical and methodological variability between studies. Instead we describe and report on the state of the published evidence to date.

## 3. Results

Mind-body therapies are briefly described in Table 1. While the therapies are described in discrete categories, considerable crossover exists between them. For example, guided imagery is often included as a part of meditation and relaxation protocols, yoga has a meditative component to it, and biofeedback for stress reduction often involves relaxation or autogenic training.

### 3.1. Search Results

**Figure 1:**
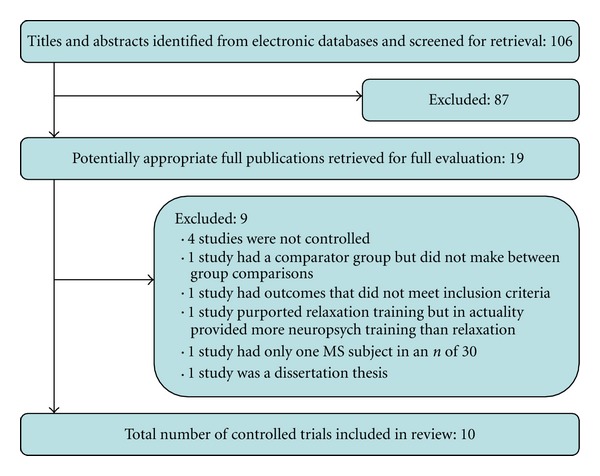
Flow diagram of literature search and study selection process.

Searching three electronic databases revealed a total of 106 articles. Titles and available abstracts were screened for clinical trials that used a mind-body intervention for MS; 87 articles were excluded because they were nonintervention papers and 19 articles were identified for further review (see Figure 1). From this, 10 studies were selected based on the inclusion criteria above. Six mind-body modalities are included in this paper: two studies of hypnosis, one of yoga, two of relaxation, two of mindfulness, two of biofeedback, and one study of guided imagery. Of the two mindfulness studies, one incorporated the principle of “mindfulness of movement” from tai chi/qi gong. We categorized this study as a mindfulness trial. No specific studies of tai chi or qi gong were identified. 

### 3.2. Description of Included Studies

A summary of the characteristics of the 10 studies is provided in Table 2. There were seven randomized controlled trials (RCTs) [25, 27, 30–34], one controlled trial in which the first eight eligible subjects were enrolled in the experimental group and the subsequent 14 subjects were randomized [28], one prospective cohort study with matched controls [26], and one prospective repeated measures design that compared within subject change between four different hypnosis interventions [29]. Subjects were recruited from local MS Society chapters, outpatient neurology clinics, community advertisements, rehabilitation centers, and local practitioners. Sample sizes varied from 20 to 150; mean sample size was 46.6 ± 40.1. In total, 466 people with MS were studied. Only three of the studies reported MS type [25, 26, 30]. Two studies used McDonald criteria [38] to confirm diagnosis [25, 27], three reported diagnosis confirmation by a neurologist but did not discuss criteria [26, 31, 32], two studies reported confirmed diagnoses but did not report by who or how [30, 33], and three studies did not report whether MS diagnosis was confirmed or not [28, 29, 34]. Five studies measured participants' baseline Expanded Disability Status Scale (EDSS) scores [25, 27, 30–32]; three of these studies provided mean EDSS scores for intervention and comparator groups. The EDSS is a measure of neurological impairment from 0 (normal neurological exam) to 10.0 (dead). Patients with scores in the 1.0–3.5 range have mild disability with no limitation of walking, 4.0–6.5 have increasing difficulty walking, and those 7.0 or higher require a wheelchair [39]. The average baseline EDSS for these five studies ranged from 2.9 to 5.9. The duration of interventions varied from four weeks (one training session a week) to 6 months (one training session a week). Assessment tools varied greatly and included a range of subjective and objective measures consisting of physical (disability, pain intensity, fatigue, incontinence, and symptom checklists), psychological (mood profiles, anxiety, pain catastrophizing, and sense of control over health), cognitive (attention, memory, and executive function), and quality of life measures. Eight studies specifically acknowledged asking participants to practice techniques on their own as homework [25, 27–30, 32–34], and six of these studies made some attempt to quantify homework adherence [25, 27, 28, 30, 32, 33]. Dropout rates and loss to follow-up ranged from 0% to 33%. 

### 3.3. Methodological Quality of Included Studies

Table 3 summarizes the risk of bias for each study. Review authors' judgments are categorized as High risk of bias (−), Unclear risk of bias (?), or Low risk of bias (+). Assessments consider the risk of bias of sufficient magnitude to have a notable impact on the results or conclusions of the trial. Several of the studies failed to provide enough detail for adequate assessment. Details regarding random sequence generation and allocation concealment were particularly poorly reported. Three studies did not provide sufficient details about baseline characteristics of comparison groups [28, 29, 31]. As it is impossible to blind participants to interventions that require their active involvement, all 10 studies received High risk of bias scores for this category. Sample sizes were generally small and seven of the studies had ≤33 subjects. Only three studies provided intention to treat analysis [25, 27, 30]. Overall, we found four of the 10 studies to be methodologically sound [25, 27, 30, 32], three to have an Unclear risk of bias [28, 33, 34], and three to have high overall risk of bias (Table 3) [26, 29, 31]. 

## 4. Discussion

The objective of this paper was to assess the published evidence for using mind-body techniques for symptom management of multiple sclerosis. Four high quality studies showed that mindfulness, yoga, biofeedback, and relaxation had a positive effect on depression, anxiety, fatigue, quality of life, and bladder incontinence (Table 4) and no effect on disability, executive function, or other cognitive measures. The remaining studies demonstrated benefit for balance and daily pain intensity, but had no effect on executive function, mood, or disability—although many methodological inadequacies were identified. This paper demonstrates that studies of mind-body techniques for treating MS symptoms are feasible, and that more stringently designed, well-executed research is needed in this population to determine efficacy. 

### 4.1. Implications for Research

More rigorous research is needed utilizing mind-body techniques for MS. Of the few studies identified in this paper, most were not of high quality, often due to small numbers and a lack of assessor blinding. In trials where it is impossible to blind participants to the study intervention, it is imperative to ensure that those assessing outcomes have no knowledge of the subjects' group assignment. Reporting on the blinding methodology for outcome assessors including maintenance of blinding throughout the study will improve the quality of future trials. 

Placebo effects due to expectation are important to assess in trials where subjects are not blinded. Because subjects know which intervention group they will be participating in, it is possible that anticipation of improvement may influence outcomes. Only one study in this paper evaluated expectation effects. Jensen et al., 2009 found that expectancy was associated with a positive treatment outcome in response to self-hypnosis training for chronic pain. It is important to measure such relationships so that expectancy effects are distinguished from true intervention effects, and the impact of expectancy on outcome can be determined. Furthermore, such research may help identify ways to enhance expectancy in the clinical setting. In clinical practice when patients are given a known therapy, the effectiveness of the therapy is a combination of nonspecific and biologically active effects. Understanding the nonspecific effects that contribute to beneficial findings in trials (e.g., expectation, self-efficacy, motivation for improvement, locus of control, patient/provider relations, and attitudes toward one's disease and healing) will further our knowledge of mind-body therapies and enhance the generalizability of research trials to clinical practice.

The selection of appropriate comparison groups is critical when designing nonblinded intervention trials using mind-body techniques. Six studies in this paper used a nonintervention control group (either usual care or waitlist) [25–27, 32–34]. A limitation of this design is that it does not control for nonspecific factors involved in the treatment group like social effects of group work and attention from instructors [40]. A well-chosen active comparison group can control both treatment-specific and nonspecific effects of the study intervention, although this kind of comparison has disadvantages as well. A drawback to active controls is that they can exert their own therapeutic benefit beyond nonspecific social effects, thus making comparisons between groups difficult to interpret [41]. For example, Oken et al. compared yoga with an active control (exercise) to evaluate yoga-specific effects and control time and attention of the intervention and found few differences between groups. Exercise is known to have a therapeutic benefit in MS [42] and this could have been problematic had the authors not also included a nonintervention (waitlist) group for comparison. Three-arm trials that include both active and nonintervention controls are favorable if they can be afforded. Challenges regarding control group selection for mind-body medicine are not new and have been described by others [41, 43, 44]. 

Level of disability should be considered during the design of a study. One example is highlighted by the Klarskov study [31]. The biofeedback protocol in this trial was specifically designed to enhance the awareness of muscles in the pelvic floor, yet 11 of the 20 subjects could not voluntarily contract pelvic floor muscles as measured by EMG, and 18 of the 20 subjects were unable to contract muscles voluntarily as measured by digital exam. The goal of the intervention was to use EMG feedback and yet a majority of the participants were unable to produce an EMG signal. Thus, the high baseline disability scores may have accounted for the absence of biofeedback effect between groups. Inclusion criteria for a baseline disability that allowed for an appropriate EMG feedback would have strengthened the study design and helped determine a therapeutic benefit. 

While it may appear self-evident that mind-body therapies have a favorable risk-benefit ratio, only one study specifically discussed safety in terms of adverse events [27]. Four papers reported why subjects dropped out of the study, but furnished no other safety information [25, 26, 30, 33]. Based on limited data, no intervention-related safety issues were identified in this paper. Even though mind-body interventions are typically gentle and serious adverse events, unlikely, future trials should collect data necessary to calculate appropriate risk assessments.

In order to advance mind-body research for MS, future studies must attempt to adequately power their trials for an appropriate sample size. Only three studies reported power analyses a priori and as a result had adequate sample sizes [25, 27, 32]. The remaining trials did not benefit from power analyses and thus risked type II error, or false negative results.

### 4.2. Implications for Practice

Mind-body therapies are valuable because they can improve symptoms that affect quality of life. For example, fatigue is one of the most common and troubling symptoms in MS. MS-related fatigue is associated with decreased quality of life and depression and is described as the worst symptom of MS by 69% of patients [45]. It is estimated that 75–95% of MS patients suffer from fatigue [45, 46] and pharmaceutical treatment provides limited relief. Our paper found that both yoga and mindfulness training improved MS fatigue, offering support with fewer side effects than conventional treatment. Therapies that can improve quality of life and the patient's ongoing experience of MS are essential to the successful management of this disease.

While there are not enough research studies to provide strong evidence for the use of mind-body therapies in MS, there is no evidence stating that they are harmful either. Patients who express interest in participating in such therapies do not necessarily need to be dissuaded because of harmful effects. However, clinicians should be mindful of a few key points when discussing mind-body medicine with MS patients.

- Hypnosis can allow distressing, repressed psychological content to come into consciousness and may not be appropriate for some patients with psychiatric disorders [47].
- Guided or motor imagery requires that individuals imagine themselves performing a task, including the sensations that may accompany that activity and may not be suitable for patients with substantial cognitive impairment [48].
- Yoga may not be appropriate for people with musculoskeletal conditions unless postures can be modified for individual circumstances. Props, such as cushions, blocks, and straps, can aid patients in holding poses, requiring less muscular effort. Bikram Yoga is practiced in a heated room (typically 105°) and risks exacerbating MS symptoms [49]. Working with a teacher who has experience adapting yoga practice for those with disabilities is advised.
- Practitioner training and licensing may be an issue for some modalities. Health care providers can use biofeedback devices within the scope of practice defined by their license. Voluntary certification is available [50] for those who do not hold a health care license, but is not currently required by most states. Thus, training and experience may vary greatly from one therapist to the next and should be investigated. Hypnosis is typically practiced as an adjunct to psychotherapy by mental health care providers; however, most states do not require a license to practice and a hypnotherapist's credentials should be investigated. Patients should be advised to seek out appropriately trained providers for the modality they are interested in.

### 4.3. Limitations of This Paper

The current study had limitations that should be considered in interpreting the results. A single author performed database searches, conducted initial reviews, and extracted data; thus the initial study selection and data extraction may have been subject to bias and error. To address this, a librarian formulated a second search strategy that failed to retrieve any new articles (see the Appendix). That both search strategies retrieved identical results suggests no bias at the initial searching level. Only English language trials were included. Including foreign language search results may have contributed additional studies. Other studies may have been inadvertently missed because a manual search of references and reviews was not done. Authors were also not contacted for missing information. Regardless of these limitations, this is the first paper to assess the published research for mind-body medicine in MS. This is a starting point upon which one can build further assessment of the efficacy and feasibility of mind-body therapies for MS. 

## 5. Conclusion

We found evidence to suggest that mind-body therapies are effective for treating common MS symptoms, including fatigue, anxiety, depression, incontinence, and quality of life. Mind-body modalities appear safe, can be prescribed as an adjunct to conventional care, and may be especially helpful when psychosocial stress is a factor or non-pharmacological options are desired (e.g., polypharmacy, pregnancy, and patient preference). More rigorously designed trials of mind-body interventions applied specifically to MS are needed in order to determine their efficacy and optimal selection for specific MS symptoms. Such research will enhance our understanding of the clinical effects of mind-body medicine as well as increase awareness and availability to clinicians and patients alike.

## Figures and Tables

**Table 1:** Description of mind-body therapies and percent use by the general public.

Modality	Description	Use by the general public (%) [10]
Meditation	(i) A mental training that is a state of being more than a task. Practices incorporate self-observation and awareness, emotional and attentional regulatory strategies, and the cultivation of an attitude of acceptance. (ii) Many forms exist, share some distinctive features but vary in purpose and technique. (iii) Most widely researched forms include transcendental meditation and mindfulness meditation. (a) Transcendental meditation: a silent word or phrase (a mantra) is repeated in order to reduce mental activity. (b) Mindfulness meditation: practitioners cultivate an open, nonjudgmental awareness of both internal experiences (thoughts, emotions, and bodily sensations) and external experiences (sights, sounds) in the present moment. Has been formalized for clinical intervention with mindfulness-based stress reduction, a program that is an amalgam of several mind-body techniques, including mindfulness meditation, breathing exercises, yoga postures, and relaxation techniques.	9.4

Yoga	(i) Incorporates physical postures, breathing, meditation into a multifaceted approach to physical/mental wellbeing. (ii) Many different practices of yoga, each varying in focus. (a) Hatha yoga is typically gentle with an emphasis on poses and breathwork. (b) Ashtanga and Vinyasa yoga are more physically demanding, moving from posture to posture without stopping. (c) Iyengar yoga is most concerned with precision of poses, encourages prop use to attain correct alignment. (d) Bikram Yoga is practiced in a heated room (typically 105°F). (e) Kundalini yoga incorporates an added emphasis on the breath in conjunction with physical poses. (iii) Like meditation, the practice of yoga cultivates a way of being rather than performing a task, although, Western practices that focus on exercise and physical health rather than awareness, insight, or spirituality have emerged.	6.1

Hypnosis	(i) Relaxed state of focused, inward attention in which peripheral awareness is reduced. (ii) Attaining alteration of consciousness involves absorption, dissociation, and suggestibility. (a) Absorption: deep immersion in an internal experience. (b) Dissociation: disconnect from peripheral events that would normally be conscious (perceptions, thoughts, emotions, or sensory activity). (c) Suggestibility: suspension of conscious editing (not asking “why?”), respond to suggestion more readily. (iii) Therapist may use guided imagery or hypnotic suggestion to help the person to understand behavioral patterns and envision making desired change. (iv) Self-hypnosis techniques can be used at home, reducing the cost of therapy and encouraging self-efficacy.	0.2

Biofeedback	(i) Electrodes placed on the body provide feedback to the patient about peripheral physiological markers like heart rate, breathing rate, muscle tension, or electrodermal activity. (ii) Neurofeedback uses scalp electrodes to measure EEG activity. (iii) Information relayed with visual or auditory cues; patient can attempt to change thoughts, emotions, or behaviors in order to control physiological reactions, such as slowing the heart rate or relaxing certain muscles. (iv) Strategies are developed and refined with a practitioner, then utilized in real time outside the therapeutic encounter. (v) Portable biofeedback devices available to enhance relaxation. (vi) Individualized treatment goals depend on the specific condition being addressed.	0.2

Relaxation	(i) Reduces reactivity to physical, psychosocial, and environmental stressors by reducing sympathetic nervous system arousal and enhancing parasympathetic response [16]. (ii) The physiological counterpart of the *fight-or-flight, *response [17]. (iii) Jacobson's progressive muscular relaxation technique and autogenic training are formalized relaxation techniques, all the mind-body therapies initiate some kind of relaxation response. (iv) Often incorporates breathing techniques that create awareness of breathing rate, rhythm, and volume. (v) Voluntary control of breathing patterns influences autonomic nervous system functions: heart rate variability, cardiac vagal tone, and CNS excitation as indicated by EEG and MRI [18]. (vi) Relaxation training enhances awareness of nervous system activation; patients can employ techniques at any time to reduce reactivity.	15.6

Imagery	(i) Most prominent forms are guided imagery and motor imagery. (a) Guided imagery: involves visualization and imagination, goal of evoking a state of relaxation or a specific outcome (visualizing the repair of myelin, or one's white blood cells attacking a tumor). (b) Motor imagery: patient relives the sensations of undertaking a skilled movement without actually doing the movement [19]. Physiologic similarities between physically executed and imagined movements have been noted in motor evoked potentials, MRI, PET, and EMG studies [20–24]. (ii) Can engage visual, tactile, kinesthetic, olfactory, gustatory, or any combination of these senses. (iii) Imagery typically developed and refined with a therapist, then practiced regularly outside the therapeutic encounter. Motor imagery is used in addition to physical therapy or exercise and not as an isolated treatment. (iv) Tailored to condition and abilities, it should be practiced in patient's own context to be meaningful for their progress.	2.2

**Table 2:** Summary of included studies.

Study	Design	Intervention versus comparison	Participant characteristics	Duration of intervention	Dropout/loss to follow up	Outcomes	Results	Overall risk of bias
Grossman et al., 2010 [25]	RCT	Mindfulness training (*n* = 76) versus usual care (*n* = 74)	RRMS or SPMS	1 session/wk × 8 wks	5%	*Primary*: quality of life (PQOLC & HAQUAMS), depression (CES-D), fatigue (MFIS). *Secondary*: anxiety (STAI).	Mindfulness training improved QOL (PQOLC *P* < .0001, HAQUAMS *P* = .0002), depression (*P* = .0002), fatigue (*P* = .0005), and anxiety (*P* = .002) when compared to usual care.	Low

Mills and Allen, 2000 [26]	Prospective cohort with matched controls	Mindfulness training (n = 12) versus usual care (n = 12)	SPMS	6 one-to-one sessions	33%	Balance (single leg stand), Symptom Rating Questionnaire (SRQ). *(No primary outcomes stated.) *	Balance improved from baseline in mindfulness group (*P* < .05) but not usual care (between group comparison not made for a balance). SRQ improved after-mindfulness and at 3-month followup compared to controls (*P* < .001).	High

Oken et al., 2004 [27]	RCT	Iyengar yoga (n = 26) versus exercise bike (n = 21) versus wait List (n = 22)	MS type not specified	Yoga: 90 min/wk for 6 months Exercise: 1 class/wk for 6 months	17%	*Primary*: attention (Stroop Color and Word Test), alertness (EEG). *Secondary*: attention (modified Useful Field of View task, adapted attentional shift task, PASAT, Wechsler Memory Scales III Logical Memory, Wechsler Adult Intelligence Scale III Similarities), alertness (SSS), mood (POMS), depression (CESD-10), anxiety (STAI), fatigue (MFI), quality of life (SF-36), disability (Timed Walk, 9-hole Peg Test, Forward Bend Flexibility).	There was no significant effect of yoga or exercise on attention, alertness, cognitive measures, mood, physical disability, or general quality of life. Both yoga and exercise reduced general fatigue (*P* < .01) and improved energy (*P* < .001) when compared to wait list controls.	Low

Jensen et al., 2009 [28]	Controlled trial	Self-hypnosis (n = 15) versus PMR (n = 7)	MS with chronic daily pain, type not specified	10 training sessions of self-hypnosis or PMR	0%	*Primary*: pain intensity (0–10 NRS). *Secondary*: pain interference (modified BPI).	Hypnosis reduced average daily pain intensity scores compared to PMR (*P* < .05). No group difference in pain interference (*P* < .10).	Unclear

Jensen et al., 2011 [29]	Prospective, repeated measures, within subject treatment comparison	Education versus self-hypnosis training (HT) versus cognitive restructuring (CR) versus self-hypnosis and cognitive restructuring (Hybrid)	22 people with MS and chronic daily pain, type not specified	4 sessions of each of the 4 interventions	32%	*Primary*: pain intensity (0–10 NRS), frequency of catastrophizing cognitions (PCS). *Secondary*: pain interference (modified BPI), worst pain intensity (0–10 NRS).	HT and Hybrid decreased pain intensity compared to pretreatment levels (*P* < .05). HT, CR, and the Hybrid reduced pain catastrophizing scores compared to pretreatment (HT *P* < .05, CR *P* < .05, Hybrid *P* < .05) but there were no differences between the three trainings. Only Hybrid reduced pain interference compared to pretreatment (*P* < .05).	High

McClurg et al., 2006 [30]	RCT	PFT (Group 1, n = 10) versus PFT & biofeedback (Group 2, n = 10) versus PFT, Biofeedback, and EStim (Group 3, n = 10)	Women with RRMS, SPMS, or PPMS	1 session/wk × 9 wks	10%	*Primary*: leakage episodes per 24 hrs (3-day diary). *Secondary*: 24 hr pad test, voiding frequency, maximum flow rate, postvoid residual volume, strength of pelvic floor muscles, quality of life (KHQ, MSQOL), incontinence impact (IIQ), urinary distress (UDI).	Leakage episodes decreased 58% (*P* = .028) in Group 2 and 76% (*P* = .003) in Group 3 compared to baseline; little change in Group 1. This reduction was significant between Groups 1 and 3 (*P* < .014). Groups 2 and 3 improved in KHQ symptom severity scale (*P* < .034), quality of life (*P* < .025), and urinary distress (*P* < .001) scales. No significant changes in pad test, voiding frequency, maximum flow rate, postvoid residuals, and pelvic floor muscle strength between groups.	Low

Klarskov et al., 1994 [31]	RCT	PFT and pharmacotherapy (n = 10) versus PFT, pharmacotherapy, and biofeedback (n = 10)	General rehabilitation in patients with MS, type not specified	Biofeedback: 30–60 min lesson q2wk, median 3 times. PFT: 40 min sessions twice a week (median 6 times).*	10%	Subjective visual analog for incontinence and voiding symptoms, leakage episodes per 24 hrs (diary), pad weight, cystometric capacity, mean voided volume, maximum flow rate, residual urine. *(No primary outcomes stated.) *	No significant differences between treatment group and controls for all outcomes. Subjective symptoms improved (*P* < .01), number of incontinence episodes decreased (*P* < .05) and maximal cystometric capacity increased (*P* < .01) for all participants. No changes seen in pad weight, mean voided volume, maximum flow rate, and residual volume for all participants.	High

Ghafari et al., 2009 [32]	Controlled trial	PMR Training (n = 35) versus usual care (n = 35)	MS type not specified	16 days of training in PMR followed by 8 weeks of home practice with CD	6%	*Primary*: quality of life (SF-8).	PMR training group showed significant improvements in quality of life compared to usual care controls (*P* < .004).	Low

Sutherland et al., 2005 [33]	RCT	Autogenic training (n = 14) versus usual care (n = 12)	MS type not specified	1 session/wk × 10 wks	15%	Quality of life (MSQOL), mood (POMS-SF), depression (CES-D). *(No primary outcomes stated.) *	AT group reported more energy than control group (*P* = .01) and were less limited in their roles due to physical and emotional problems (*P* = .02, *P* = .05). No significant differences between groups were seen for mood or depression.	Unclear

Maguire, 1996 [34]	RCT	Imagery (n = 15) versus usual care (n = 18)	MS type not specified	Six 1-hour group sessions	0%	Mood (POMS), anxiety (STAI), health attribution (HAT), MS symptom checklist (developed by PI). *(No primary outcomes stated.) *	Imagery group showed no change in mood compared to controls. Imagery showed decrease in state anxiety after intervention (*P* < .05) but not trait anxiety compared to controls. The “internal control of health” score for imagery group remained stable but decreased for controls (*P* < .05).	Unclear

BPI: Brief Pain Inventory; CES-D: Center for Epidemiologic Studies-Depression Scale; EStim: Neuromuscular electrical stimulation; HAQUAMS: Hamburg Quality of Life Questionnaire in Multiple Sclerosis; HAT: Health Attribution Test; IIQ: Incontinence Impact Questionnaire; KHQ: King's Health Questionnaire; MFI: Multidimensional Fatigue Inventory; MFIS: Modified Fatigue Inventory Scale; MSQOL: MS Quality of Life Instrument; NRS: Number Rating Scale; PASAT: Paced Auditory Serial Addition Test; PCS: Pain Catastrophizing Scale; PFT: pelvic floor training; PMR: progressive muscle relaxation; POMS-SF: Profile of Mood States-Short Form; PPMS: primary progressive MS; PQOLC: Profile of Health-Related Quality of Life in Chronic Disorders; RCT: randomized controlled trial; RRMS: relapsing remitting MS; SPMS: secondary progressive MS; SSS: Stanford Sleepiness Scale; STAI: State Trait Anxiety Inventory; UDI: Urinary Distress Inventory.

*Total number of sessions over what length of time is unclear.

**Table 3:** Summary of risk of bias assessment.

Study	Intervention	Random sequence generation	Allocation concealment	Blinding of participants and personnel	Blinding of outcome assessment	Incomplete outcome data	Selective reporting	MS diagnosis and criteria	Other bias*	Overall risk of bias for study
Grossman et al. 2010, [25]	Mindfulness	+	+	−	+	+	+	+	+	+
Mills and Allen, 2000 [26]	Mindfulness	?	?	−	−	−	?	+	−	−
Oken et al., 2004 [27]	Yoga	+	+	−	+	+	+	+	?	+
McClurg et al., 2006 [30]	Biofeedback	+	?	−	+	+	+	+	+	+
Klarskov et al., 1994 [31]	Biofeedback	?	?	−	−	+	+	?	−	−
Jensen et al., 2011 [29]	Hypnosis	?	?	−	+	−	+	?	−	−
Jensen et al., 2009 [28]	Hypnosis	?	?	−	+	+	+	?	−	?
Ghafari et al., 2009 [32]	Relaxation	?	?	−	?	+	+	+	+	+
Sutherland et al., 2005 [33]	Relaxation	?	?	−	?	+	+	+	−	?
Maguire, 1996 [34]	Imagery	?	?	−	?	+	+	?	−	?

(+) Low risk of bias.

(−) High risk of bias.

(?) Unclear risk of bias.

*Other bias category includes small sample size (*n* < 30) [35], studies which reported being underpowered and minimal or no group comparison at baseline.

**Table 4:** Of 10 studies reviewed, four high quality studies were found helpful for symptoms of MS.

Study	Intervention	Helpful for
Ghafari et al., 2009 [32]	Relaxation	Quality of Life

Grossman et al., 2010 [25]	Mindfulness-based stress reduction	Depression Anxiety Fatigue Quality of Life

Oken et al., 2004 [27]	Yoga	Fatigue

McClurg et al., 2006 [30]	Biofeedback	Bladder incontinence

## Appendix

Two search strategies were applied to each electronic database: MEDLINE, PsycINFO, and Cochrane Clinical Trials Register.

1. Hannon search strategy: (exp Mind-Body Therapies OR exp Mind-Body Relations, Metaphysical) *AND* (exp therapeutics OR th.fs.) *AND* exp multiple sclerosis OR multiple sclerosis, chronic progressive OR multiple sclerosis, relapsing-remitting).
2. Senders search strategy: (exp multiple sclerosis OR multiple sclerosis, chronic progressive OR multiple sclerosis, relapsing-remitting) *AND* (qi gong.mp. OR exp Meditation OR mindfulness.mp. OR exp Relaxation OR exp Muscle Relaxation OR exp Relaxation Therapy OR exp Breathing Exercises OR breathing technique.mp. OR breathwork.mp. OR breathwork.mp. OR exp Hypnosis OR exp “Imagery (Psychotherapy)” OR exp Yoga OR exp Biofeedback, Psychology OR exp Tai Ji OR tai chi.mp.).
